# Numerical Investigation of the Fetal Left Heart Hemodynamics During Gestational Stages

**DOI:** 10.3389/fphys.2021.731428

**Published:** 2021-09-09

**Authors:** Huseyin Enes Salman, Reema Yousef Kamal, Huseyin Cagatay Yalcin

**Affiliations:** ^1^Department of Mechanical Engineering, TOBB University of Economics and Technology, Ankara, Turkey; ^2^Pediatric Cardiology Division, Hamad General Hospital, Hamad Medical Corporation, Doha, Qatar; ^3^Biomedical Research Center, Qatar University, Doha, Qatar

**Keywords:** congenital heart defects, fetal heart analysis, computational fluid dynamics, wall shear stress, hemodynamics, mitral valve, left heart

## Abstract

Flow-driven hemodynamic forces on the cardiac tissues have critical importance, and have a significant role in the proper development of the heart. These mechanobiological mechanisms govern the cellular responses for the growth and remodeling of the heart, where the altered hemodynamic environment is believed to be a major factor that is leading to congenital heart defects (CHDs). In order to investigate the mechanobiological development of the normal and diseased hearts, identification of the blood flow patterns and wall shear stresses (WSS) on these tissues are required for an accurate hemodynamic assessment. In this study, we focus on the left heart hemodynamics of the human fetuses throughout the gestational stages. Computational fetal left heart models are created for the healthy fetuses using the ultrasound images at various gestational weeks. Realistic inflow boundary conditions are implemented in the models using the Doppler ultrasound measurements for resolving the specific blood flow waveforms in the mitral valve. Obtained results indicate that WSS and vorticity levels in the fetal left heart decrease with the development of the fetus. The maximum WSS around the mitral valve is determined around 36 Pa at the gestational week of 16. This maximum WSS decreases to 11 Pa at the gestational week of 27, indicating nearly three-times reduction in the peak shear stress. These findings reveal the highly dynamic nature of the left heart hemodynamics throughout the development of the human fetus and shed light into the relevance of hemodynamic environment and development of CHDs.

## Introduction

Congenital heart defects (CHDs) affect a wide range of society around the globe with a prevalence of 1.0–1.2% (Hoffman, 2013). These defects include improper growth of the ventricles, arteries, and valves (Hoffman and Kaplan, 2002), and they initiate in the early stages of the fetal heart development. The exact mechanism and etiology of CHDs remain unclear (Yalcin et al., 2011). Even though genetic factors play a major role in the formation of CHDs (Zaidi et al., 2013), they are not the only source for these defects. It is reported that only 2–4% of CHDs are observed in families with prior CHD history, showing that most of the cases are observed in infants with no prior history of disease in their families (Øyen et al., 2009). This fact indicates that the mechanobiological mechanisms are also important in the formation of CHDs. Hemodynamic forces such as the blood flow driven shear stresses are critical parameters that can influence the proper development of the endothelial and endocardial tissues (Salman et al., 2019; Salman and Yalcin, 2020), because these biomechanical factors orchestrate the responses of the cells which are responsible for the cardiac growth and remodeling during the developmental stage (Sedmera et al., 1999; Reckova et al., 2003; Groenendijk Bianca et al., 2005; deAlmeida et al., 2007).

The hemodynamic development in the left heart is particularly important because the left heart provides the required pumping power for the blood circulation. The CHDs observed in the left heart such as hypoplastic left heart syndrome (HLHS) result in underdeveloped left heart which cannot sustain the systemic circulation (Sedmera et al., 1999; Kowalski et al., 2014; Lindsey et al., 2014). For the defected fetal left hearts, the blood flow is directed to the right side of the heart due to the underdeveloped left side. As a result, the biomechanical environment and the shear stress distribution in the heart are disrupted in the presence of CHDs. The quantification of the altered flow hemodynamics through the clinical measurements is required to clearly understand the differences between the healthy and defected cases. However, clinical *in vivo* flow measurements and hemodynamic assessments are quite challenging for the fetal hearts (Chen et al., 2017). The Doppler ultrasound measurements can only resolve the average blood flow velocities at specific planes within the heart chambers or vessels. Experimental *in vitro* studies also contain many oversimplifications in the fetal heart models which do not completely represent the *in vivo* flow conditions. Therefore, an accurate clinical modality is needed to elucidate the complex hemodynamic parameters inside the entire heart for monitoring the changes in the flow behavior during the cardiac development.

At this point, computational fluid dynamics (CFD) modeling is a beneficial tool which enables to model and analyze the entire heart considering the realistic flow conditions (Bharadwaj et al., 2012; Lindsey et al., 2015; Olcay et al., 2018). CFD modeling is widely used for the investigations in the cardiovascular research (Pekkan et al., 2005; Yalcin et al., 2017). It is based on solving the governing fluid flow equations in a spatially and temporally discretized domain. As consequence, the detailed flow analyses and hemodynamic assessments offer a deep understanding of CHDs, and accurate assessments can be performed using CFD modeling approach in order to investigate the altered flow behavior during the development of the fetal heart.

In the literature, there are a limited number of CFD studies investigating the cardiovascular flow in the human fetus, mainly due to the challenges in acquiring the fetal heart geometry and measuring the realistic inflow conditions (Pennati et al., 2008; Saw et al., 2017). In several computational studies, the volumetric contraction of the left (Lai et al., 2016) and right ventricles (Wiputra et al., 2016; Zebhi et al., 2020) are simulated for determining the amount of flow in the fetal heart.

In this study, we investigated the left heart hemodynamics of the healthy human fetal hearts. Various gestational stages, ranging from week 16 to week 27, are modeled using 2D geometric models based on ultrasound imaging to monitor the alterations in flow dynamics during the heart development. CFD simulations revealed the complex hemodynamics and shear stress environment around the mitral valve. The behavior of evolving flow-driven parameters is investigated to understand the biomechanical environment in the healthy fetal hearts during the developmental stages.

## Materials and Methods

Computational fluid dynamics modeling approach is employed for resolving the complex flow fields in the left side of the fetal heart. The flow geometry is determined using the ultrasound based medical images. The governing fluid flow equations are solved using commercial finite element analysis software ANSYS Fluent (Canonsburg, PA, United States). The realistic boundary conditions are clinically measured and applied to the generated models for increasing the accuracy of the hemodynamic assessments. Critical parameters such as blood flow velocity, pressure, and wall shear stress (WSS) are elucidated to monitor the alterations in the left heart throughout the fetal development.

### Model Geometry

**FIGURE 1:**
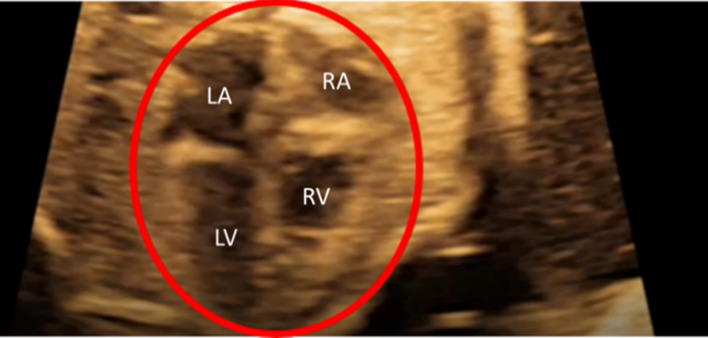
Sample medical image of a fetal heart which is clearly indicating the four heart chambers. This example is for a 26-week fetal heart. LA is left atria, LV is left ventricle, RA is right atria, and RV is right ventricle.

Ultrasound images of healthy prenatal fetal hearts are used to determine the left ventricle and left atrium geometries. The borders of four heart chambers are clearly visible in the selected fetal hearts as shown in Figure 1. Due to the lack of images at multiple layers of the heart, we were capable to generate 2D (two-dimensional) geometric models for the prenatal fetal hearts. Five different healthy (control) hearts are investigated to reveal the hemodynamics at different gestational stages. Control 1 heart is investigated at two distinct gestational stages, at week 16 and week 24. Control 2, Control 3, and Control 4 hearts are investigated at only one gestational stage, which are week 25, week 26, and week 19, respectively. Control 5 heart is also investigated at two different gestational stages, which are week 19 and week 27. Therefore, our hemodynamic assessment covers the gestational development within the week 16 and week 27.

**FIGURE 2:**
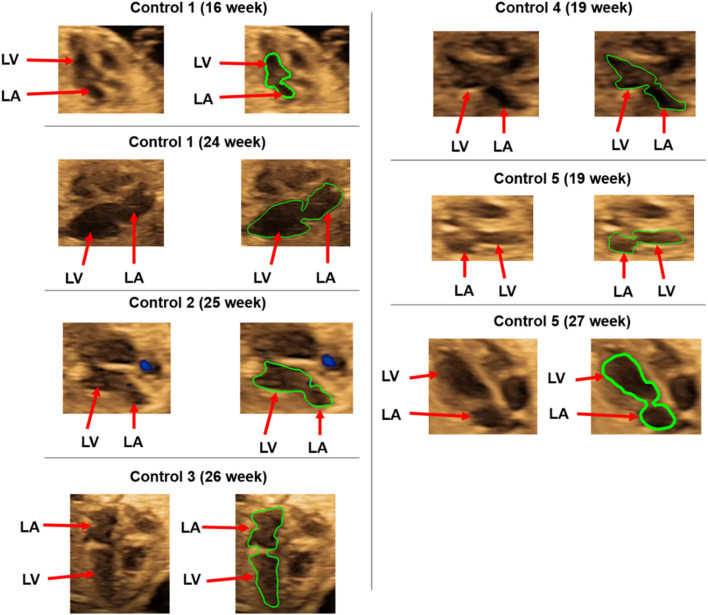
Control hearts used for the CFD analysis. The boundaries of the left ventricle (LV) and left atrium (LA) are shown in green.

**FIGURE 3:**
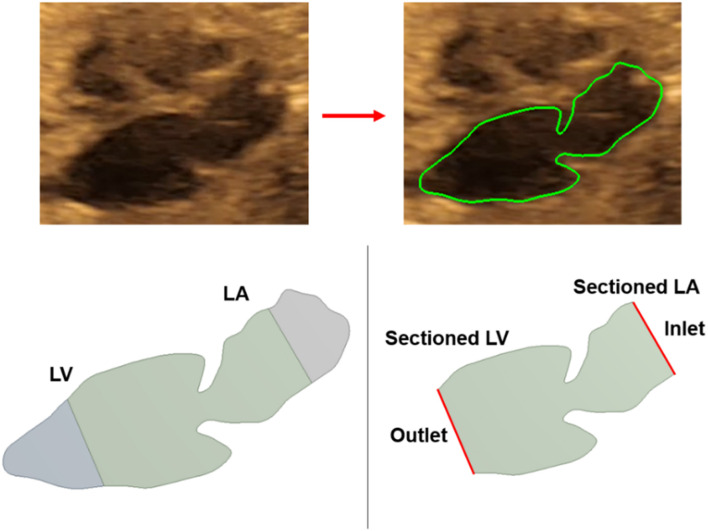
Medical image of a sample fetal heart and defining the boundary layers. About one third of the left ventricle and left atrium are removed from the outer edges. The inlet and outlet flow boundaries are defined at the sectioned regions.

For each of the control hearts, the borders of the left atrium and left ventricle are determined as presented in Figure 2. In reality, these borders are dynamic, and their shapes continuously change depending on the contraction of the chambers during the cardiac cycle. The mitral valve is located between the left atrium and left ventricle, and serves as a bridge between these two heart chambers. During the left atrium contraction, the edges of the atrium more distant from the mitral valve move toward the mitral valve to compress the chamber and divert the flow toward the left ventricle. In other words, the edges close to the mitral valve experience less contraction and they are relatively static than the far sides of the chamber. After the contraction of the left atrium, the blood is directed and filled into the left ventricle which leads to a volumetric increase in the ventricle. In the same manner, the edges of the ventricle distal to the mitral valve undergo greater deformation, and edges close to the mitral valve are less deformed during the blood filling period. Therefore, the regions close to the mitral valve are relatively stationary during the cardiac cycle. Considering these movement dynamics of the cardiac chambers, we limited our analysis to the proximity of the mitral valve. The sides of the left atrium and left ventricle close to the mitral valve are included in the analysis, but the highly deformed parts of the left ventricle and left atrium are removed due to the highly dynamic edges of the chambers which are exposed to high deformations during the contraction and blood filling. One-third of the chambers are removed from the outer edges as shown in Figure 3. This way, we simplified the contraction and blood filling processes in the chambers and used the relatively motionless parts of the chambers in the CFD models. Therefore, our static CFD models accurately capture the left ventricular filling where the mitral valve is fully open and the left ventricle is fully dilated. The predicted results of CFD simulations are expected to be most accurate only during the left ventricular filling phase, when the mitral valve is completely open and the adjacent chamber geometries do not change significantly. It is worth to note that if the CFD results of the static geometries employed in the current study are examined during the left ventricular systolic phase, where the left ventricle ejects blood from the fully open aortic valve into the aorta, the numerical findings would be inaccurate and prone to error due to neglecting the highly dynamic geometrical changes in the heart chambers. Therefore, the results of the current investigation are presented within 0–0.25 s which corresponds to the diastolic left ventricular filling.

### Boundary Conditions

**FIGURE 4:**
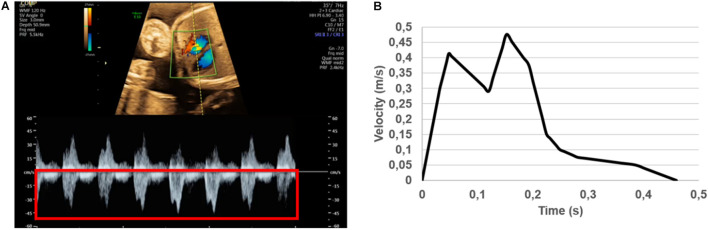
**(A)** Doppler ultrasound measurements to determine the blood flow velocity waveform in the mitral valve. The red rectangle shows multiple cardiac cycles. The average of multiple cardiac cycles is used to define the inlet flow conditions. **(B)** Numerically plotted blood velocity waveform at the mitral valve during one cardiac cycle for the case studied in **(A)**. For each of the control heart, clinically measured specific inlet waveform is used to prescribe the inlet flow velocity of the CFD simulations.

**FIGURE 5:**
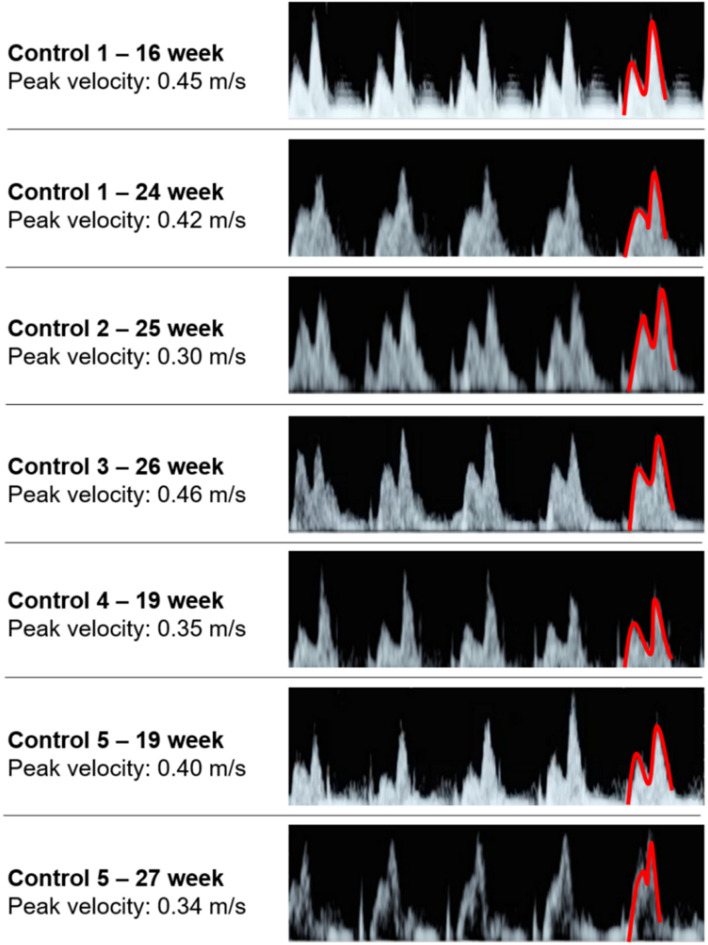
Five sequential mitral valve flow waveforms measured using Doppler ultrasound for each fetal heart. The first half of the last waveform is highlighted in red, which is showing the local maximum and peak flow velocities.

**FIGURE 6:**
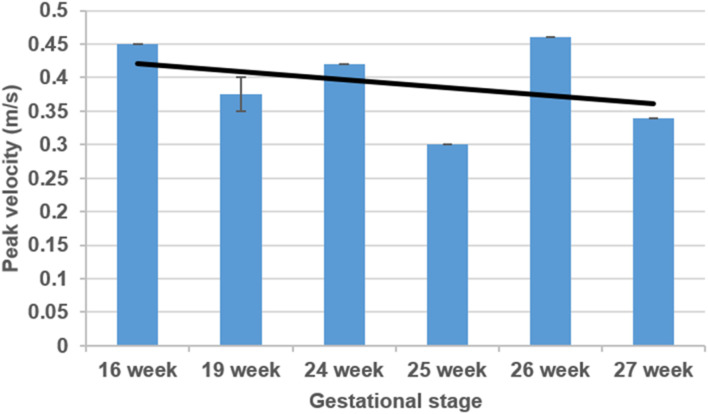
Comparison of mitral valve peak flow velocities considering various gestational stages. The black line is determined by curve fitting using linear regression.

The sectioned edges of the chambers are set as inlet and outlet flow boundaries as given in Figure 3. We clinically measured the flow profiles in the mitral valves using Doppler ultrasound measurements. An exemplary clinical Doppler flow measurement in the mitral valve is presented in Figure 4A. The waveform provided in the red rectangle is the blood flow velocity in the mitral valve as function of time. This waveform is numerically plotted in Figure 4B. For each gestational stage, five sequential waveforms are used to determine the average flow waveform in the mitral valve as shown in Figure 5. This averaged mitral valve flow waveform is employed in the CFD analysis by applying a numerically scaled version of this waveform at the inlet flow boundary. For each of the fetal hearts, a unique flow waveform is applied as the inlet boundary condition depending on the clinical ultrasound measurements. The mitral valve flow waveforms are similar for the investigated fetal hearts with a local maximum followed by a peak flow velocity. The main difference between the waveforms is related to the peak flow velocities. In Figure 6, the peak flow velocities in the mitral valve are compared for various gestational stages. There is only one sample heart for the weeks 16, 24, 25, 26, and 27 and two samples for week 19. A straight line is fitted for the peak mitral valve velocities using linear regression. According to the fitted line, the peak mitral valve velocity slightly decreases depending on the increasing gestational stage. The expected peak mitral valve velocities are around 0.425 and 0.36 m/s at week 16 and week 27, respectively. The peak of the Doppler ultrasound measurement at week 26 is observed around 0.46 m/s which is considered as an exceptional case. The general trend indicates a reduction in the flow velocities with the development of mitral valves.

The inlet velocity profile of the CFD model is adjusted to satisfy the clinically averaged flow velocities in the mitral valve. By manipulating the inlet velocity amplitude on the inlet flow boundary, the clinically measured mitral valve velocities are achieved in our CFD models. For the manipulation process, first we apply a random inlet velocity in the CFD model and calculate the corresponding mitral valve velocity using the CFD results. Then, we gradually change the inlet flow rate in the CFD model until we reach the same mitral valve flow rates for both the clinical Doppler ultrasound measurements and CFD results. After trial and error, the required inlet flow waveform is determined as function of time to achieve the clinically measured mitral valve flow rate in the mitral valve of the CFD model. This way, it is guaranteed that the mitral valve flow velocities of the CFD models are the same with the clinical mitral valve flow measurements. For each of the control heart, the same procedure is repeated at the inlet flow boundary using the unique mitral valve waveforms for each different case. On the outlet flow boundary, the gage pressure is set as zero and the rest of the boundaries are set as wall boundaries with no slip condition. The inlet, outlet, and wall boundaries are assumed as rigid walls with no deformation. Therefore, the flow driven hemodynamic forces do not cause any change in the model geometry.

### Governing Equations

The continuity and Navier–Stokes equations are solved to determine the flow parameters. Considering a homogeneous and incompressible fluid medium, the continuity and Navier–Stokes equations can be described as given in Eqs 1, 2, respectively (Scotti and Finol, 2007).

(1) ∇⋅v=0

(2) $$\rho \,\frac{\partial \text{v}}{\partial t}+\rho \,\left(v\right)\cdot \nabla \text{v}-\nabla \cdot \tau =\text{f}$$

In Eq. 1, **v** defines the fluid velocity vector in the flow domain. The continuity equation guarantees that the mass flow rate at the inlet and outlet boundaries are equal, and the total mass in the flow domain is preserved. In Eq. 2, ρ denotes the fluid mass density, *t* denotes time, τ denotes the fluid stress tensor, and **f** denotes the vector of body forces. The effect of gravity is negligible on the flow velocities, therefore the body forces term (**f**) is set to zero. The Navier–Stokes equation is based on the conservation of momentum, mass, and energy. The fluid stress tensor (τ) can be written in terms of the fluid pressure (*p*), as given in Eq. 3 (Scotti et al., 2008).

(3) $$\tau =-p\,\delta_{\mathrm{ij}}+2\,\mu \,\varepsilon_{\mathrm{ij}}$$

In Eq. 3, δ_*ij*_ defines the Kronecker delta, μ defines the dynamic viscosity of the fluid, and *ε*_*ij*_ defines the strain rate. The strain rate can be expressed in the form of the fluid velocity vector (**v**) as given in Eq. 4.

(4) $$\varepsilon_{i\,j}=\frac{1}{2}\,\left(\nabla \text{v}+\nabla {\text{v}}^{\text{T}}\right)$$

### Flow Model

For the gestational stages within 16–27 weeks, the heart beat rate is reported around 130 beats per minute (Pildner von Steinburg et al., 2013), which corresponds to an approximate cardiac cycle length of 0.46 s. Among the control hearts, there are small variations in the cardiac cycle length which is less than 5%. For the ease of comparison, we normalized all cardiac cycle lengths to 0.46 s. For each of the control hearts, the transient flow analysis is performed for one cardiac cycle using 46 time steps with 0.01 s time increments. The blood is modeled as a Newtonian fluid with a constant viscosity of 0.0035 Pa s (Roldán-Alzate et al., 2014) and mass density of 1060 kg/m^3^ (Bove et al., 2003). In the control hearts, the maximum Reynolds number at the mitral valve reaches approximately 400 and this value is much lower than the turbulence transition limit which is specified as the Reynolds number of 2000 (Eckhardt et al., 2006). Since the maximum Reynolds numbers of the control hearts are significantly less than the turbulence transition limit, a laminar flow model is employed in the CFD analysis. Considering the incompressible nature of the flow, a pressure-based solver is used for solving the governing flow equations, and a second order upwind scheme is employed for the discretization of momentum equations (Amindari et al., 2017). The heat transfer is neglected in the CFD models due to the indiscernible effect on the flow parameters.

### Mesh Independence

**FIGURE 7:**
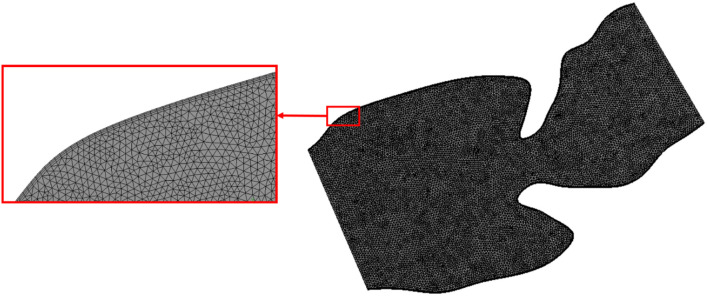
The mesh with moderate density for the sectioned control 1 heart at week 24. Mesh density is increased on the wall boundaries as shown in the red box for improving the solution accuracy.

After defining the geometry and assigning the boundary conditions, computational meshes are generated by discretizing the flow domain into a finite number of spatial elements. In order to have mesh independent results, three different mesh densities are employed for each analysis. The coarse, moderate, and dense meshes are composed of approximately 8,000, 20,000, and 50,000 triangular elements. There are three velocity nodes and one pressure node at each triangular element. The results of meshes are compared for a sample case considering the control 1 heart at week 16. The area weighted average vorticity through one cardiac cycle is determined as 108.28, 126.21, and 127.12 s^–1^ for the coarse, moderate, and dense meshes, respectively. There is a 16.55% difference of area weighted average vorticity between the results of the coarse and moderate meshes. This difference is decreased to 0.73% between the moderate and dense meshes. Therefore, the results of the moderate mesh are found as satisfactorily accurate since the difference between the moderate and dense meshes is less than 2% (Kelsey et al., 2017). In order to improve the accuracy of the results, the density of mesh is increased around the wall boundaries by adding inflation layers as shown in Figure 7. This way, the formation of boundary layers near the walls are monitored more accurately.

## Results

Results are obtained for five healthy control hearts without any CHDs. Two of the control hearts are analyzed at two different gestational stages, and other hearts are analyzed at only one gestational stage. The analyses cover the range of week 16 and week 27. Mainly investigated parameters are the flow velocity, pressure, vorticity, and WSS.

**FIGURE 8:**
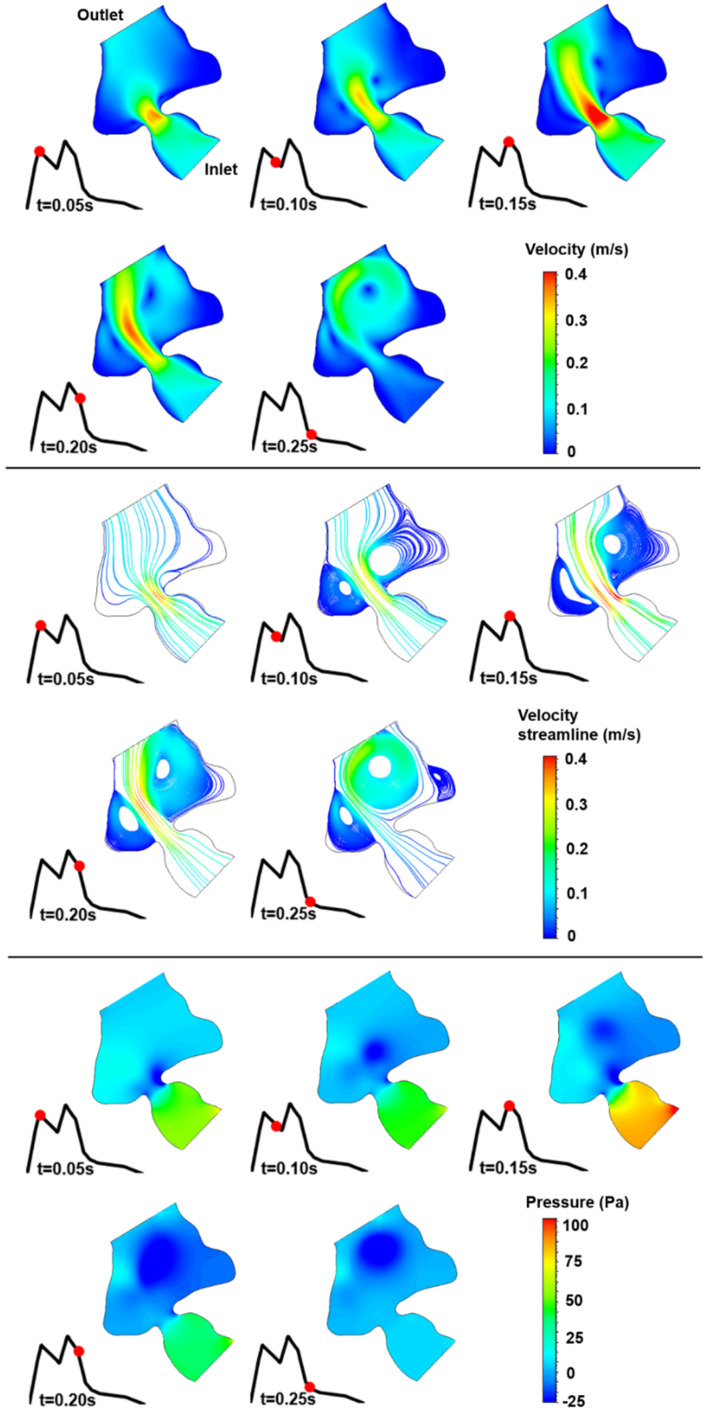
Velocity magnitude, velocity streamline, and pressure contours for the control 1 heart at week 16. The instant in the cardiac cycle is shown near the plots.

**FIGURE 9:**
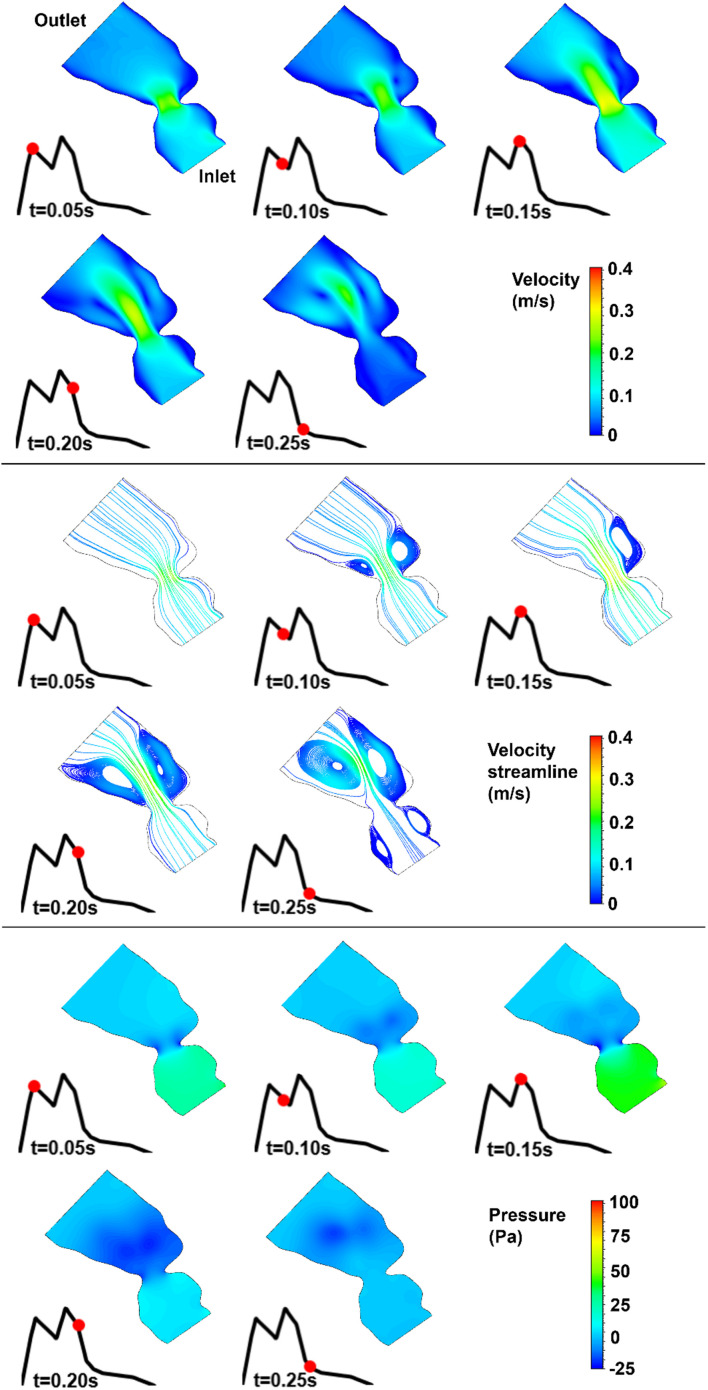
Velocity magnitude, velocity streamline, and pressure contours for the control 2 heart at week 25. The instant in the cardiac cycle is shown near the plots.

**FIGURE 10:**
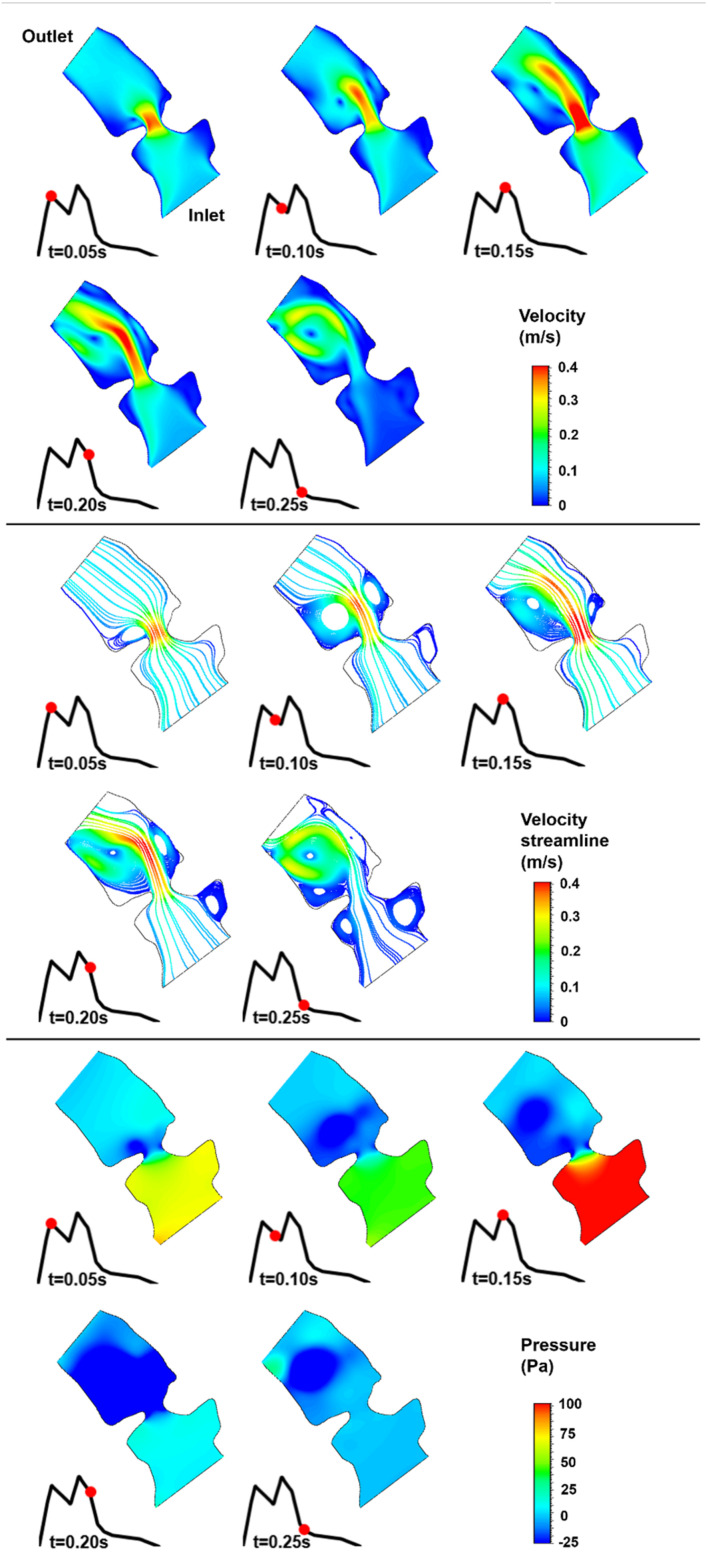
Velocity magnitude, velocity streamline, and pressure contours for the control 3 heart at week 26. The instant in the cardiac cycle is shown near the plots.

**FIGURE 11:**
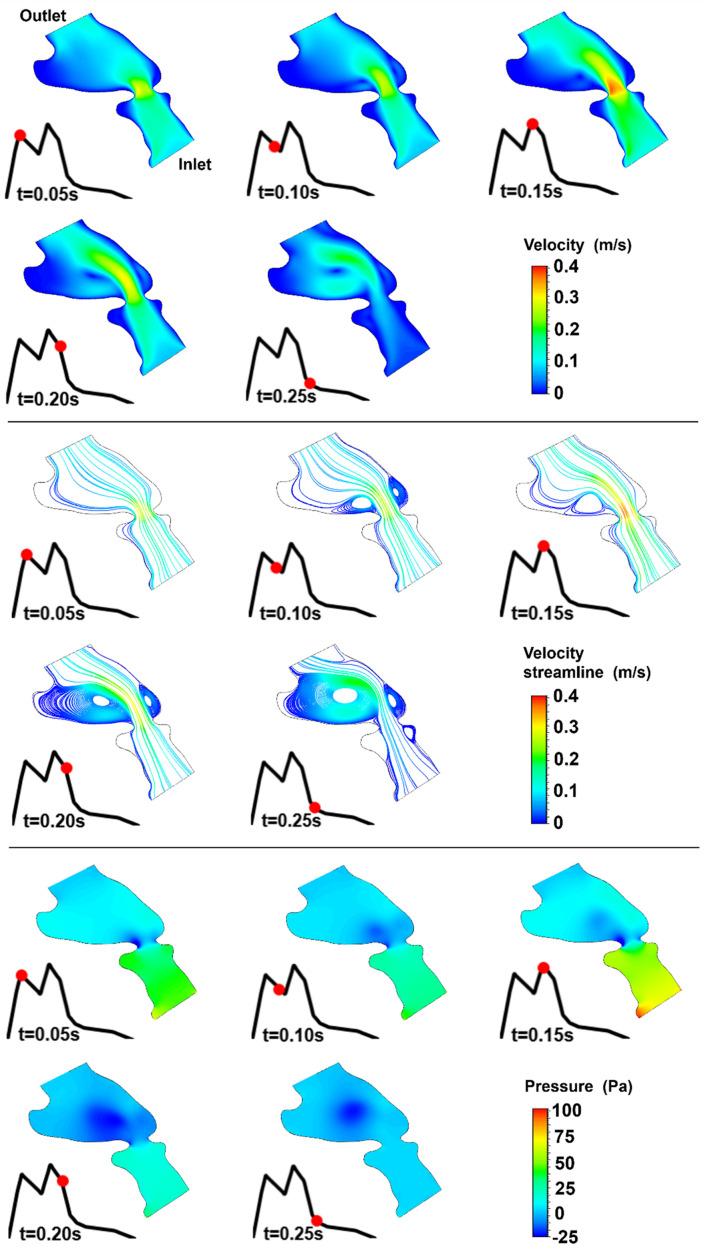
Velocity magnitude, velocity streamline, and pressure contours for the control 4 heart at week 19. The instant in the cardiac cycle is shown near the plots.

**FIGURE 12:**
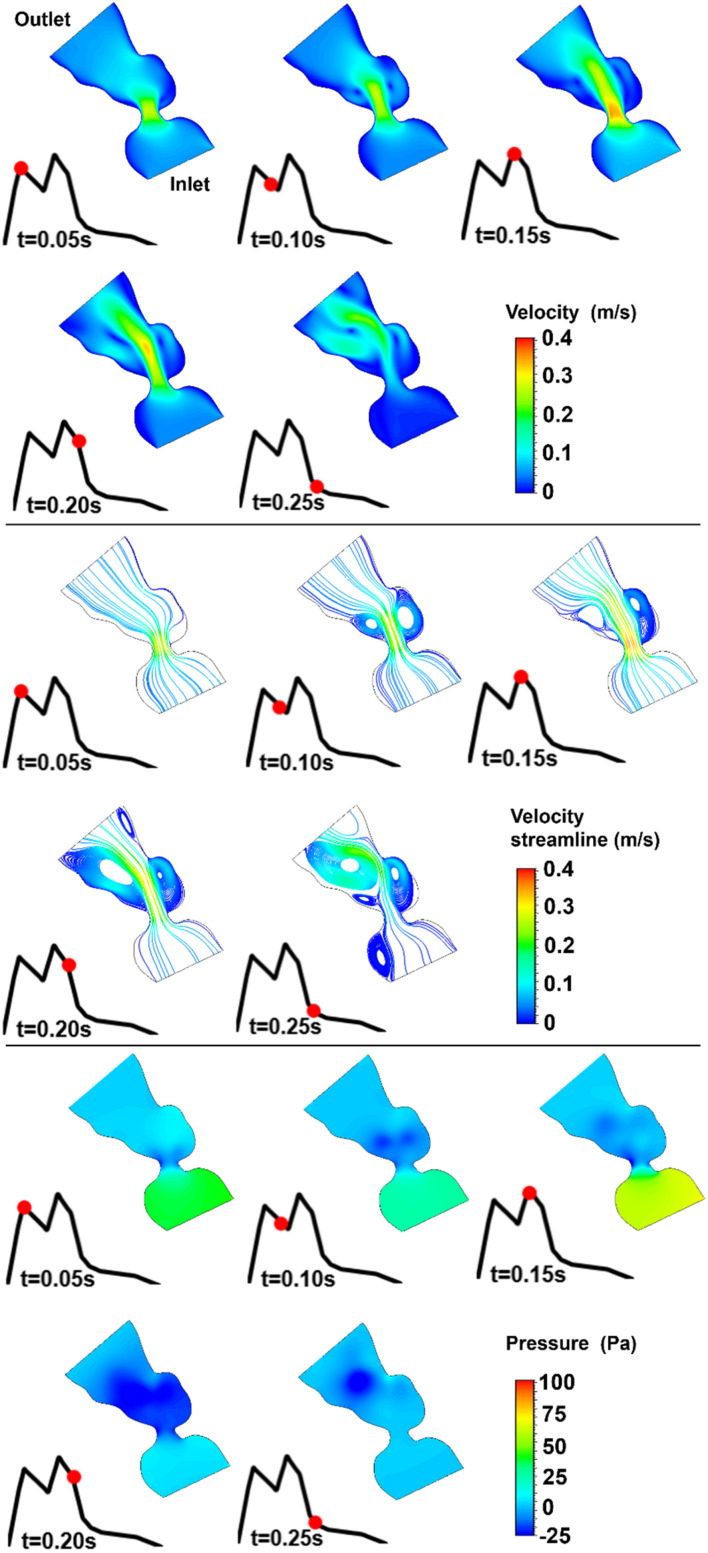
Velocity magnitude, velocity streamline, and pressure contours for the control 5 heart at week 27. The instant in the cardiac cycle is shown near the plots.

The blood flow velocities, flow streamlines, and pressure contours are plotted in Figures 8, 9, 10, 11, 12 for the control 1, 2, 3, 4, and 5 hearts, respectively. The inlet and outlet boundaries of the control hearts are shown in each figure. The same color scales are used in the figures for the ease of comparison.

For all of the control hearts, the peak flow velocity is observed around 0.15 s. A high-velocity flow jet is formed in the mitral valve, and the flow velocity slowly scales down through the end of the cardiac cycle. The peak flow velocities in the fetal hearts are ranged between 0.325 and 0.475 m/s. After the peak flow rate is observed, recirculating vortices appear especially in the left ventricle and maintain until the end of the cardiac cycle. For all of the fetal hearts, one primary vortex is observed in the left ventricle. The total number of recirculating vortices increase with the curved shape and geometric complexity of the left ventricle.

The pressure difference between the inlet and outlet boundaries has the maximum value at the instant of peak flow rate. The difference in the inlet and outlet pressures gradually decreases until the end of the cardiac cycle. The peak value of the pressure difference is ranged within 60–100 Pa for the control fetal hearts. A region with negative pressure is observed at the center of the primary recirculating vortex, which acts as a suction source in the left ventricle. It is observed that the magnitude of the negative pressure can reach −25 Pa.

**FIGURE 13:**
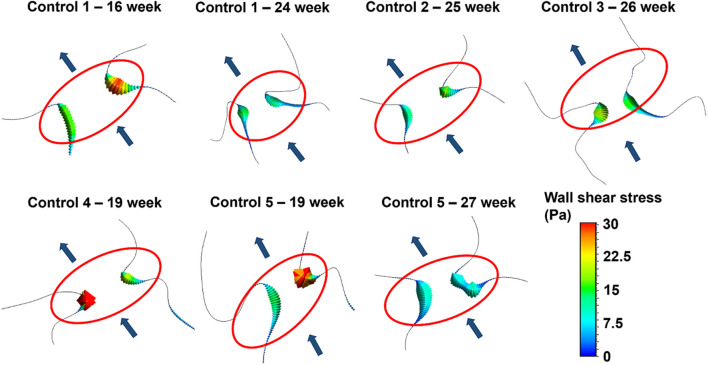
Peak WSS levels on the two sides of the mitral valve at different gestational stages. The flow direction is shown by the arrows. The proximity of the mitral valve is shown inside the red ellipse.

The magnitude and distribution of WSS on the mitral valve have critical importance. Because, the altered shear stresses may deteriorate the proper growth and remodeling of the endothelial cells and this condition may initiate congenital defects. For this purpose, WSS levels are investigated on the two sides of the mitral valve. In Figure 13, the peak WSS levels in the mitral valve are presented for all control hearts. The area shown in the red ellipse is the proximity of the mitral valve which is exposed to high shear stresses.

According to the results presented in Figure 13, the WSS levels on the two sides of the mitral valve are significantly different at the early gestational stages between the week 16 and week 19. One side of the mitral valve is exposed to much higher WSS level around 30 Pa. Interestingly, a relatively regular WSS distribution is observed around the mitral valve as the fetal heart develops. The WSS levels become similar for the two sides of the mitral valve between the week 24 and week 27, where the peak WSS is around 15 Pa. This value is approximately half of the peak WSS observed in the early gestational stages between the week 16 and week 19.

**FIGURE 14:**
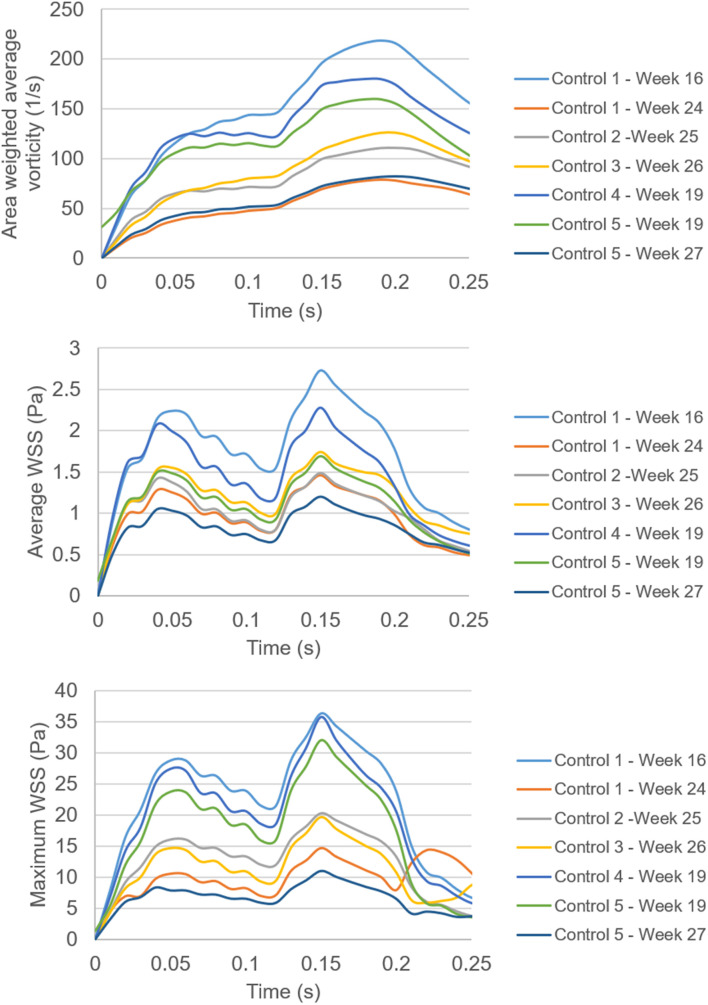
Comparison of the area weighted average vorticity in the flow domain, the average WSS around the mitral valve, and the maximum WSS around the mitral valve. Spatially averaged values of WSS are determined by averaging the WSS levels across the entire wall of the models. Spatially averaged vorticity levels are determined by averaging the values over the entire flow area in the models.

In Figure 14, the area weighted average vorticity in the flow domain, the average WSS around the mitral valve, and the maximum WSS around the mitral valve are presented as function of time. The results show that the temporal change in vorticity has a common behavior for different gestational stages and fetal hearts. The highest vorticity is observed around 0.2 s for all cases. The main difference in the vorticity plots is the different magnitude scales changing with the fetal development. This fact is also observed in the results of the average and maximum WSS levels. For different fetal heart models, the peak WSS level is observed at nearly the same instant in the cardiac cycle. Only one peak value is observed in the vorticity plots. On the other hand, for the average and maximum WSS plots, there are two distinct peak values that are closely related to the applied inlet velocity waveform.

**FIGURE 15:**
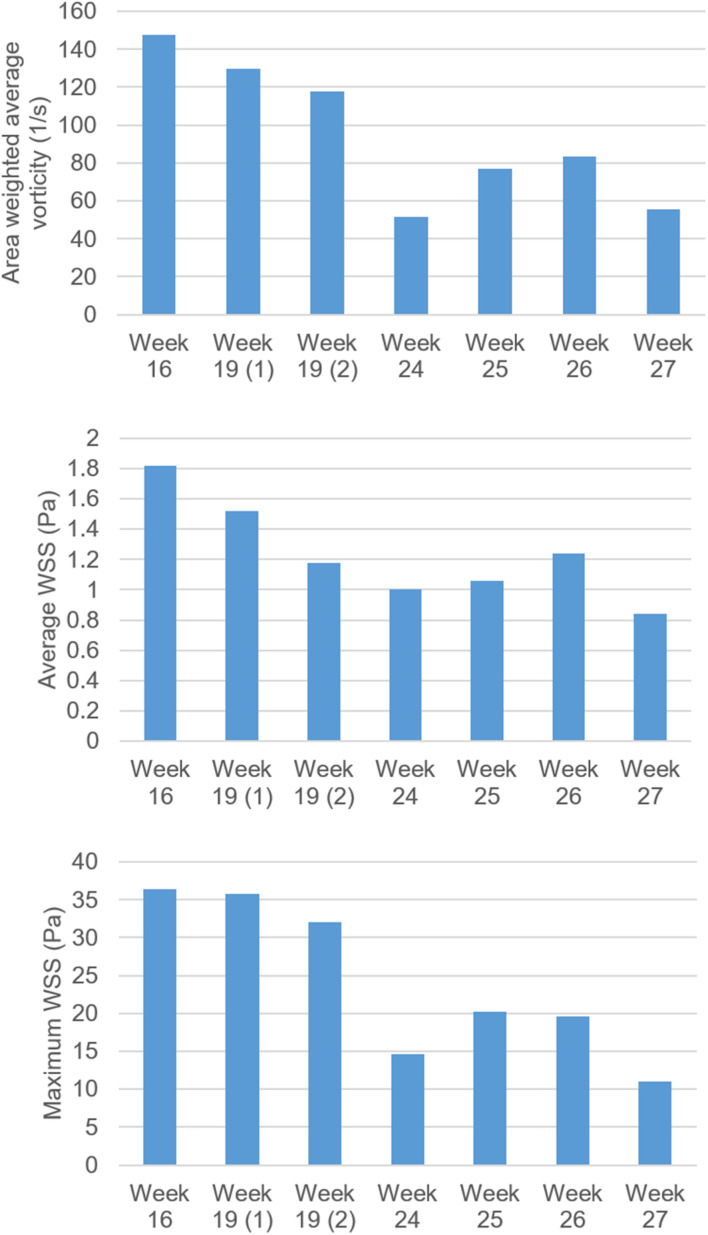
Comparison of vorticity, average WSS and maximum WSS levels for fetal control hearts by weeks of gestation. The maximum WSS is observed on the mitral valve. There are two fetal heart analyses at week 19, which are shown as 19 (1) and 19 (2). For other gestational weeks, there is only one fetal heart analysis. The area weighted average vorticity and average WSS levels are determined by averaging the values in the flow domain during 0–0.25 s, corresponding to the first half of the cardiac cycle.

In Figure 15, temporally averaged vorticity and WSS levels are presented for different weeks of gestation. These average values are determined by averaging the results over the first half of the cardiac cycle which covers 0–0.25 s. There are two different control hearts analyzed at week 19. Other control hearts are analyzed at the gestational weeks of 16, 24, 25, 26, and 27. At week 16, the average vorticity is higher than 140 s^–1^. At week 19, the average vorticity is determined around 120 s^–1^. When the week 27 is investigated, the average vorticity is determined around 60 s^–1^, which is much lower compared to the vorticity levels observed at the early gestational weeks. The results obtained indicate a significant reduction in vorticity depending on the heart development. At week 24, there is an average vorticity level of 50 s^–1^, but this value is considered to be an exceptional result. The general trend shows a certain decrease in the vorticity levels with the development of the fetus. Similarly, the average WSS levels around the mitral valve also decrease with the increasing gestational weeks. The average and maximum WSS levels at week 16 are determined around 1.8 and 36 Pa, respectively. At week 27, the average and maximum WSS levels are observed around 0.85 and 11 Pa, respectively, indicating approximately twofold reduction in the average WSS and threefold reduction in the maximum WSS.

It should be noted that the peak WSS levels are observed on the mitral valve. The levels of peak WSS may include errors in terms of magnitudes, since determining the exact dimension of the fully opened mitral valve is challenging. A small dimensional change in the mitral valve may alter the peak WSS levels. Therefore, the average WSS level around the mitral valve is a more accurate parameter to compare the results between different fetal hearts. The trend of WSS decline with the fetal development is observed in both the peak WSS and average WSS evaluations, but the assessment of average WSS is considered to be more reliable than the peak WSS on the mitral valve.

## Discussion

The assessment of hemodynamics is crucial to elucidate the biomechanical environment in the developing fetal hearts. Blood flow velocity, vorticity, and WSS levels are significantly important since these flow parameters affect the proper development of the heart chambers. CFD is a useful approach to examine the hemodynamics in a developing heart. For an accurate CFD analysis, medical image-based geometries and realistic boundary conditions are required to be implemented. This way, it is possible to numerically investigate the flow in the entire heart and determine the parameters which are critical for the heart development.

In this study, we investigated the evolving hemodynamics acting around the mitral valve in the left heart for the developing human fetuses, because many CHDs are associated with the left heart and the mitral valve. B-mode ultrasound images of fetal hearts and Doppler ultrasound velocity measurements at the mitral valve are used for the CFD models covering week 16 to week 27 of gestation. In the modeled fetal hearts, clinically measured mitral valve flow waveforms are similar in terms of the general behavior, with a local maximum and a peak in the waveform, and the peak flow velocity barely reaches 0.4 m/s around the developing mitral valve. It is observed that the fetal hearts at different gestational stages with unique left heart geometries have similar levels of valve jet velocity. On the other hand, vorticity and WSS levels are significantly influenced by the week of gestation due to the developing heart. The vorticity is an indicator of turbulent activity where the higher levels of vorticity demonstrate the characteristics of a chaotic flow. The general trend of the area weighted average vorticity and WSS shows a systematic decrease in magnitudes depending on the fetal heart development. Indeed, this indicates that the reduction of WSS and vorticity is basically governed by the volumetric increase of the left heart. As the left heart volume increases, the WSS and vorticity magnitudes tend to decrease. These results suggest that in the early stages, high levels of WSS and vorticity influence the enlargement of the left heart and the development of the mitral valve leaflets, whereas in the later stages, the enlargement of the left heart stabilizes the WSS and vorticity levels. These results are consistent with the findings in the literature (Wiputra et al., 2018). It is stated that the aorta grows by adapting to flow conditions to maintain and stabilize a certain level of WSS in the fetal development (Struijk et al., 2005). Wiputra et al. (2018) reported a time-averaged WSS in the range of 0.74–0.86 Pa in the left ventricle of normal fetuses at week 22. In our analyses, we determined an average WSS of 1.0 Pa around the mitral valve at week 24. These levels of average WSS in the left heart are in well agreement with the findings of Wiputra et al. (2018). Since we concentrate on the region around the mitral valve rather than the entire left ventricle, our WSS findings are slightly higher than the results of Wiputra et al. (2018).

The cardiac cells responsible for the growth and remodeling in the heart are quite sensitive to the shear stress levels, therefore the disturbed hemodynamics and abnormal shear stress levels influence the proper development progress (Franck et al., 2013). In case of a CHD in the left heart, the reduced flow rate due to the limited contraction of the underdeveloped left heart cannot generate the adequate levels of shear stress on the cardiac tissues. As a result, the lowered WSS levels may also initiate further defects due to the altered biomechanical environment.

When the WSS levels at two sides of the mitral valve are investigated, it is seen that there is an unbalance of shear stress at the early gestational stage. At week 16, the ratio of peak WSS at two sides of the mitral valve is determined as 1.73, which means that one side of the valve is exposed to nearly 1.7-times higher shear stress. At the later stages of gestational development, this WSS unbalance between two sides of the mitral valve tends to decrease. At week 27, the peak WSS ratio between the two sides of the valve is determined as 1.02, indicating that nearly identical peak WSS levels are experienced on both sides of the mitral valve.

There are several limitations in our study. Only the healthy fetal hearts are investigated to monitor the alterations in the flow parameters depending on the gestational stage. The investigation of fetal hearts with congenital defects would reveal the cues of the CHD formation by comparing the flow parameters in the healthy and defected hearts.

The employed CFD geometries are static and mainly consider the flow dynamics during the left ventricular filling phase, in which the mitral valve is completely open, and the left atrium and left ventricle geometries around the mitral valve are relatively stagnant compared to the outer edges of the heart chambers. This implies that our numerical predictions are only accurate during the diastolic left ventricular filling phase and the investigation does not include the hemodynamics during the left ventricular systolic phase due to neglecting the highly dynamic nature of the heart chambers.

The use of 2D geometric models is a limitation of the current investigation. Since the turbulent activity and vortex formation are inherently 3D effects, the use of 3D geometric models would improve the accuracy of the prediction of the vortex formation. The 2D models can provide reliable results at a particular region in the heart and within a limited period in the cardiac cycle. On the other hand, the use of 3D models that realistically deform over time can provide reliable results for the whole heart geometry throughout the entire cardiac cycle, taking into account the large 3D deformations in the heart chambers. The findings of the current study focus on a particular region around the proximity of the mitral valve within the ventricular filling period. This study is considered as a first step toward characterizing the fetal cardiac hemodynamics using static 2D geometries.

We have limited data for the fetal human hearts to be included at each gestational stage, which prevented to perform a detailed statistical analysis. Except week 19, only one sample heart could be examined for the gestational weeks. Only for week 19, two sample hearts are analyzed. In further studies, it is aimed to increase the number of sample hearts in order to reduce the error depending on the extraction of the problem geometry. When the two sample hearts at week 19 are compared, the relative differences in the area weighted vorticity, averaged WSS, and maximum WSS are determined as 9.4, 22.6, and 10.5%, respectively. This variance shows that the increased number of sample hearts is necessary to increase the accuracy of the average results.

The validation of the CFD results could only be performed by comparing the magnitude of the peak flow velocities in the mitral valve. The clinically measured peak velocity in the mitral valve is the same with the peak flow velocity in the numerical models. The *in vivo* determination of the entire flow domain in a developing heart is a difficult task; therefore, the entire flow fields could not be validated using the clinical tools. Since the peak flow velocities are similar both in the clinical measurements and numerical findings, we expect a realistic flow field around the proximity of the mitral valve. Since some of the 2D ultrasound images of the fetal hearts are blurry, the determination of the exact chamber borders and valve protrusions is challenging and the generated 2D geometric models can be prone to error. In further studies, it is aimed to increase the number of investigated fetal hearts in order to compensate the errors that may occur during the geometrical model generation. Instead of using 2D geometric models, generation of more realistic 3D heart models is aimed in further studies using the multiple layers of ultrasound images. The static wall boundaries can be replaced by dynamic and deformable cardiac tissues to model the developing heart more realistically.

In future studies, we also aim to elucidate the fetal hearts with CHDs by employing CFD simulations using the medical image based realistic models. In the current study, the laminar flow model is employed considering the low Reynolds number in the flow domain. The inclusion of a turbulent model and employing non-Newtonian blood flow properties can further improve the accuracy of the hemodynamic assessments. In the CFD models, blood viscosity is modeled at a constant value of 0.0035 Pa s for all weeks of gestation; however, the viscosity can be modeled as a function of the gestational stage to determine more accurate WSS levels in the numerical analysis (Christensen et al., 2014). Nevertheless, it is considered that the main conclusions of the current study are reasonably accurate around the mitral valve proximity during the diastolic ventricular filling phase and provide an insight about the hemodynamic assessment of the flow parameters depending on the gestational stages and their roles on the development of the healthy human fetal hearts.

## Conclusion

In this article, we investigated the left heart hemodynamics of the healthy human fetal hearts using 2D CFD models. The realistic inflow conditions are clinically measured and applied to the computational models. The gestational stages within the week 16 and week 27 are analyzed using five different fetal control hearts. It is concluded that the hemodynamic environment and WSS levels around the mitral valve significantly change with the development of the fetal heart. The WSS levels on the mitral valve gradually decrease with the increasing gestational weeks, mainly due to the enlargement of the heart. At the early gestational stages between week 16 and week 19, there is a WSS unbalance between the two sides of the mitral valve. At the later stages of gestation between week 24 and week 27, the growth of the mitral valve provides a relatively uniform distribution of shear stress around the valve proximity. It is concluded that the sudden changes in the average and peak WSS levels may deteriorate the growth and remodeling of the cardiac cells and lead to initiation of CHDs. In future studies, it is aimed to compare the flow parameters in healthy fetal hearts with the altered hemodynamic parameters in fetal hearts with CHDs.

## Data Availability Statement

The original contributions presented in the study are included in the article/supplementary material, further inquiries can be directed to the corresponding author/s.

## Ethics Statement

The studies involving human participants were reviewed and approved by the Hamad Medical Corporation IRB Committee and Qatar University IRB Committee. The patients/participants provided their written informed consent to participate in this study.

## Author Contributions

HS wrote the first draft and revised the manuscript. RK and HY structured, reviewed, and revised the manuscript. All authors read and approved the submitted version of the manuscript.

## Conflict of Interest

The authors declare that the research was conducted in the absence of any commercial or financial relationships that could be construed as a potential conflict of interest.

## Publisher’s Note

All claims expressed in this article are solely those of the authors and do not necessarily represent those of their affiliated organizations, or those of the publisher, the editors and the reviewers. Any product that may be evaluated in this article, or claim that may be made by its manufacturer, is not guaranteed or endorsed by the publisher.
